# Coronary Computed Tomography Angiography Assessment of High-Risk Plaques in Predicting Acute Coronary Syndrome

**DOI:** 10.3389/fcvm.2021.743538

**Published:** 2021-10-01

**Authors:** Guanyu Lu, Weitao Ye, Jiehao Ou, Xinyun Li, Zekun Tan, Tingyu Li, Hui Liu

**Affiliations:** ^1^Department of Radiology, Guangdong Provincial People's Hospital, Guangdong Academy of Medical Sciences, Guangzhou, China; ^2^College of Medicine, Shantou University, Shantou, China

**Keywords:** coronary computed tomography angiography (CCTA), high-risk plaque, acute coronary syndrome (ACS), computational fluid dynamics–CFD, pericoronary adipose tissue attenuation, coronary artery

## Abstract

Coronary computed tomography angiography (CCTA) is a comprehensive, non-invasive and cost-effective imaging assessment approach, which can provide the ability to identify the characteristics and morphology of high-risk atherosclerotic plaques associated with acute coronary syndrome (ACS). The development of CCTA and latest advances in emerging technologies, such as computational fluid dynamics (CFD), have made it possible not only to identify the morphological characteristics of high-risk plaques non-invasively, but also to assess the hemodynamic parameters, the environment surrounding coronaries and so on, which may help to predict the risk of ACS. In this review, we present how CCTA was used to characterize the composition and morphology of high-risk plaques prone to ACS and the current role of CCTA, including emerging CCTA technologies, advanced analysis, and characterization techniques in prognosticating the occurrence of ACS.

## Introduction

Acute coronary syndrome (ACS) may be the first manifestation of coronary artery disease (CAD), mainly caused by the rupture or erosion of unstable plaques (1–4), which is the leading cause of death for most of the world's population. Therefore, it has been a driving force to identify these high-risk plaques prone to rupture which may lead to ACS. Substantial study efforts have confirmed that virtual histology intravascular ultrasound (IVUS) or optical coherence tomography (OCT) can be valuable (5).

However, these invasive diagnostic approaches with low positive predictive value and unclear cost-effectiveness have not been widely used in clinical practice (6). Coronary computed tomography angiography (CCTA), as a comprehensive non-invasive imaging assessment approach, which allows for the quantification and characterization of coronary atherosclerosis, can effectively evaluate the condition of all coronary arteries and the branches in the whole-heart, and has great clinical application value in identifying adverse plaque characteristics (7, 8). The high accuracy and high efficiency of CCTA are well-confirmed in previous studies, as well as its higher diagnostic performance compared with invasive reference standards (7, 9, 10). CCTA has been used to identify the characteristics and morphology of high-risk atherosclerotic plaques associated with ACS in previous studies, including positive remodeling, low attenuation, spotty calcification, and the napkin-ring sign (8, 10, 11). The development of CCTA and latest advances in emerging technologies, such as computational fluid dynamics (CFD), have made it possible to simultaneously perform the comprehensive evaluation of anatomical severity degree, lesion geometry, plaque characteristics, quantification of hemodynamic parameters and detection of vascular inflammation, which may help to identify the high-risk plaques and predict the risk of ACS (12).

In this review, we delineate the current understanding of the pathology of the atherosclerotic plaques associated with ACS and corresponding manifestations on CCTA in clinical practice. The application of CCTA in characterizing the composition and morphology of high-risk plaques prone to ACS and prognosticating the occurrence of ACS are further described. Finally, the progress in emerging CCTA technologies, advanced analysis and characterization techniques are also reviewed. The new techniques can provide the comprehensive assessment of high-risk plaques and surrounding environment, and may provide personalized risk assessment of future ACS events and further guide clinical decision-making.

## From High-Risk Plaque Histopathology to CCTA

The detection and characterization of plaque by CCTA is based on histopathology, therefore, and therefore it is vital to grasp the histopathological characteristics and evolution process of high-risk atherosclerotic plaque.

Atherosclerosis, most often observed at the branch points and low shear stress areas of blood vessels, is a multifactorial systemic disease and has a chronic and progressive process (13). About two-thirds of ACS results from the rupture of atherosclerotic plaque (14, 15). The early manifestations of atherosclerosis are non-atherosclerotic intimal lesions, which include intimal thickening and xanthoma. Subsequently, starting from pathological intimal thickening, they further develop into increasingly vulnerable and rupture-prone lesions, and progress into fibroatheroma or even thin cap fibroatheroma, which is considered to be the precursor of plaque rupture (16). Moreover, the development of lesions before rupture can be explained by the histopathological nature of unstable lesions, including intraplaque hemorrhage, neo-vascularization, plaque healing, and recurrent rupture (17, 18). Compared with plaque erosion and stable CAD, intraplaque hemorrhage is the most common finding in plaque rupture, which includes numerous foam cells (lipid-laden macrophage), cholesterol clefts, and an expanding necrotic core, contributing to the vulnerability of plaque (19).

**Figure 1:**
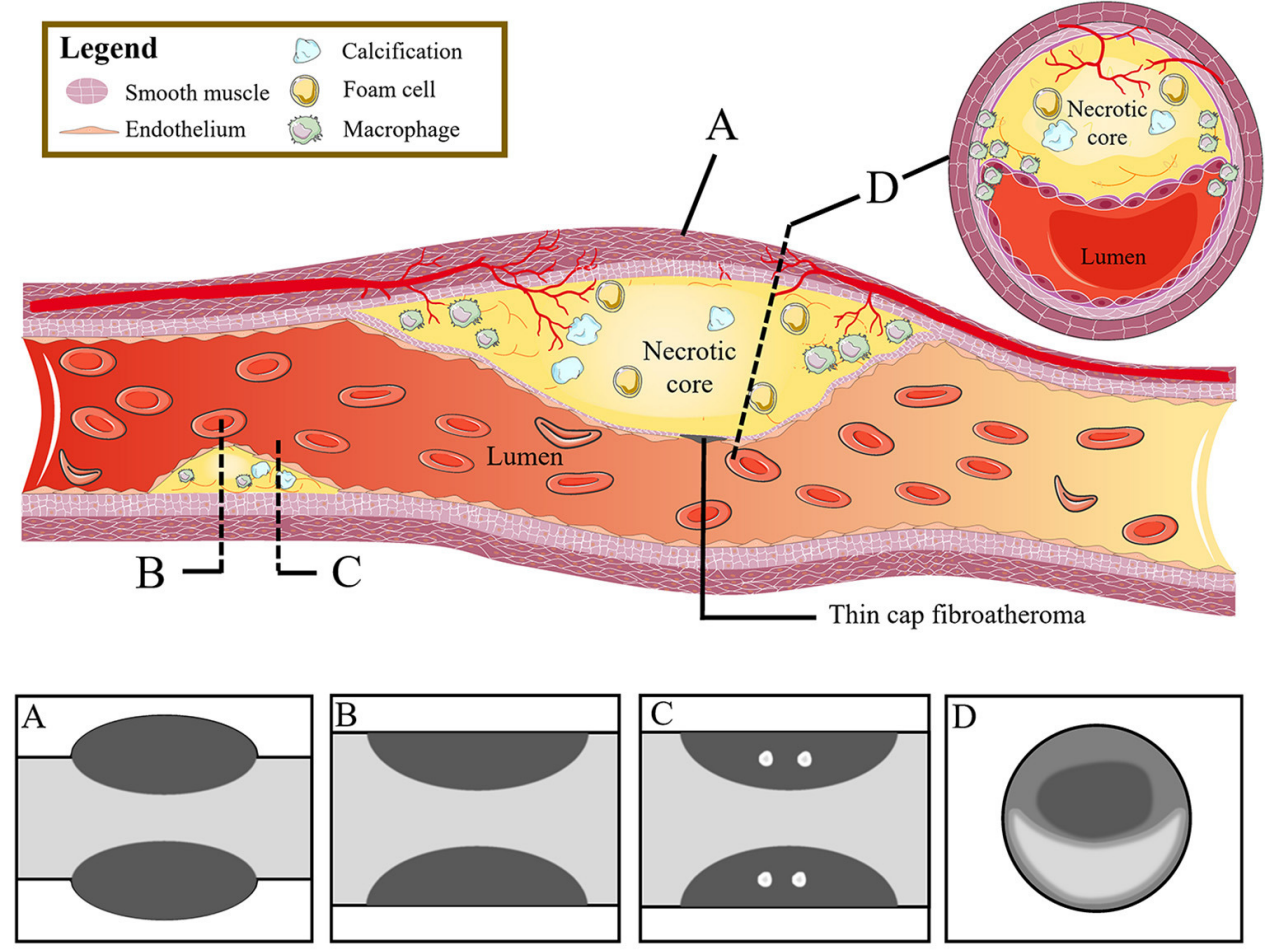
From high-risk plaque histopathology to CCTA. The figure shows the histopathologic components of vulnerable plaques (colored illustrations) and the corresponding typical CT features of high-risk plaques: **(A)** positive remodeling, **(B)** low attenuation, **(C)** spotty calcification, and **(D)** napkin-ring sign. CCTA, coronary computed tomography angiography.

The characteristics of high-risk plaques are related to the vulnerability of plaques (18). Based on the histopathologic composition of vulnerable plaques, including thin cap fibroatheroma, macrophage infiltration, and necrotic core (20, 21), the corresponding typical manifestations of high-risk plaques in CCTA are listed as follows: positive remodeling, low attenuation, spotty calcification, and napkin-ring sign (22–24) (Figure 1).

## From High-Risk Plaque CCTA Features to Acute Coronary Syndrome

### CCTA Assessment of High-Risk Plaque Features

The percentage of stenosis is important information on CCTA. When coronary heart disease cannot be ruled out clinically, the percentage of stenosis on CCTA can help to rule out patients in stable condition and with low possibility of coronary heart disease (25–27). However, the main cause of ACS is plaque rupture and erosion rather than fixed stenosis. Given that plaque instability and plaque progression are important factors leading to subsequent acute coronary events, identifying high-risk plaque characteristics is necessary (17, 25, 28–31). As a comprehensive non-invasive imaging assessment approach, CCTA has been used to identify the morphological characteristics of high-risk plaques prone to rupture that may lead to ACS (32–35).

#### Positive Remodeling

**Figure 2:**
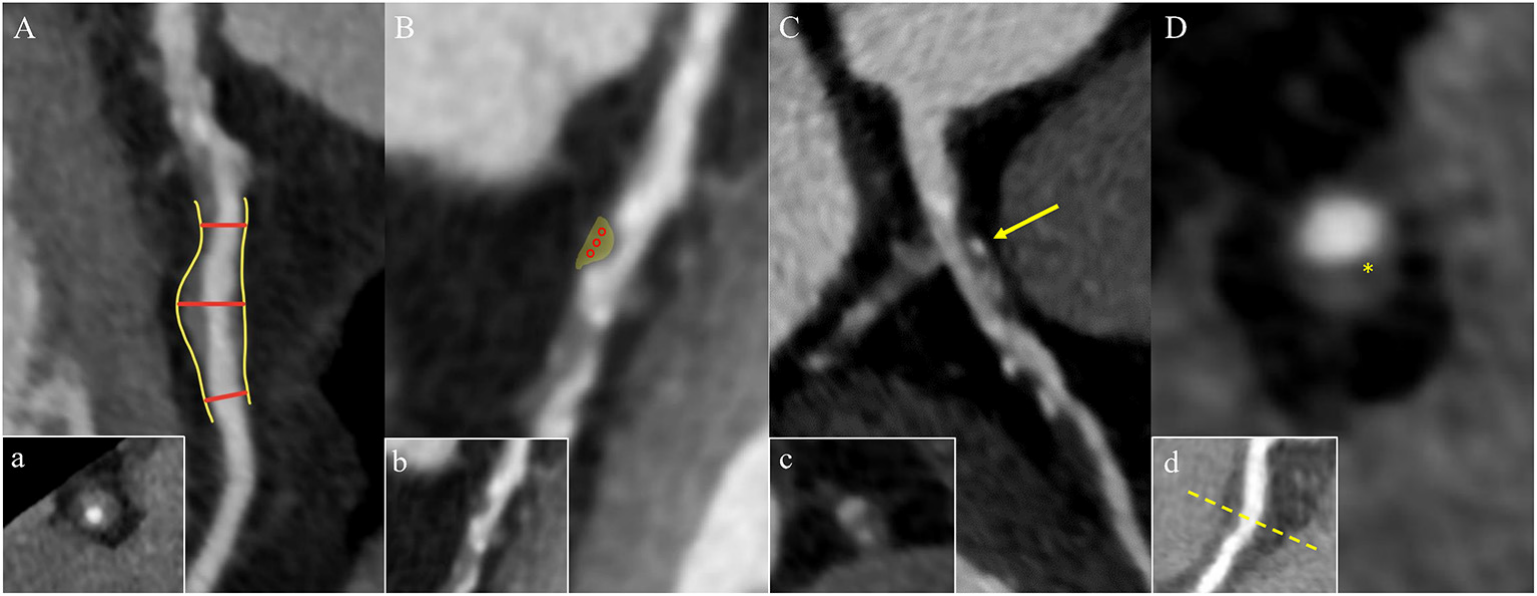
High-risk plaque characteristics on CCTA. **(A)** Positive remodeling of a non-calcified plaque in the proximal left anterior descending coronary artery. The two short red lines indicate the vessel diameters of the proximal and distal of the plaque (both 1.0 mm), and the long red line indicates the maximum vessel diameter in the middle of the plaque (1.6 mm). The remodeling index is 1.6. Picture a is the cross section of picture **(A)**. **(B)** A low-attenuation plaque (yellow area) in the mid segment of the left anterior descending coronary artery with a mean CT attenuation value of 21 HU in the three regions of interest (red circles). Picture b is the original picture **(B)**. **(C)** Spotty calcification of a partially calcified plaque surrounded by non-calcified components in the proximal left anterior descending coronary artery with a diameter <3 mm in all directions (yellow arrow). Picture c is the cross section of picture **(C)**. **(D)** A napkin-ring sign plaque in the proximal right coronary artery. The yellow star shows the central area of the plaque with a low HU close to the lumen, which is surrounded by the peripheral edge of higher CT attenuation. Picture d is the multiplanar reconstructed image of picture **(D)**. CCTA, coronary computed tomography angiography; HU, Hounsfield units.

Clinically, coronary artery remodeling refers to be the compensatory changes of cross-sectional area and structure of coronary artery in the progression of coronary atherosclerosis. In pathological findings, the lumen of some coronary arteries was found to be increased during atherogenesis in autopsy (36). For *in vivo* detection of coronary artery, IVUS examination confirmed that the cross-sectional area of the vessel at the atherosclerotic site was significantly larger than that at the proximal reference segment, then the concept of positive remodeling was proposed which refers to the compensatory increase of vessel wall when atherosclerotic plaque volume increases continuously, thus maintaining the effective area in the lumen (37). While on CT, the outer vessel wall dimension could be measured. The remodeling index (RI) is calculated by dividing the vessel cross-sectional area/diameter of the largest stenosis (or maximum vessel area/diameter) by the average cross-sectional area/diameter of the proximal and distal reference segments (7, 38, 39) (Figure 2Aa). At present, positive remodeling is generally defined as RI ≥ 1.1 in CCTA (8, 40, 41), while some researchers prefer other cut-off point (42, 43). In addition, automatic software makes it easier to quantify the RI (44).

#### Low Attenuation

The composition of plaques can be reflected by CT attenuation value, with the highest CT attenuation value for calcification, followed by fibrous tissue, and the lowest CT value for lipid. Low-attenuation plaques refer to those with the lowest CT attenuation value and the most easily ruptured lipid composition (a lipid-rich necrotic core), which is defined as mean attenuation <30 Hounsfield units (HU) of at least three regions of interest (ROIs) in general (39, 45) (Figure 2Bb). However, the CT attenuation value of lipid plaques overlaps with that of fibrous plaques, so it is difficult to distinguish the plaques only by CT attenuation value alone. In addition, the CT value of plaques is affected by many factors, such as contrast agent, plaque volume, slice thickness, tube voltage, and so on. Therefore, the current research mainly relies on special procedures to identify which plaque is low-attenuation (7, 45).

#### Spotty Calcification

Spotty calcification is the initial state of calcification. Since calcification is one of the consequences of local inflammation, spotty calcification may indicate active local inflammation. Mechanical stimulation on fibrous cap caused by spotty calcification and local inflammation may lead to the plaque with spotty calcification easy to rupture, thereby accelerating disease progression (46–48). Therefore, spotty calcification is considered to be one of the characteristics of high-risk plaques. In CCTA, spotty calcification is generally manifested as calcification in plaques with a density of more than 130 HU and a diameter of <3 mm surrounded by non-calcified components (42, 49, 50) (Figure 2Cc). However, only calcification more than 0.5 mm in diameter is visible on CT, so nearly two-thirds of the calcifications cannot be recognized on CT (51, 52).

#### Napkin-Ring Sign

The napkin ring sign is a qualitative plaque feature that can be defined by the presence of two features on the cross section of non-calcified plaques: the low-attenuation central area obviously contacting with the coronary artery lumen and the annular high attenuation plaque tissue surrounding the central area (7, 34) (Figure 2Dd). Histologically, the low-density area corresponds to the large necrotic nucleus, while the “annular” outer area is associated with fibrous tissue. The necrotic core area in plaque with the napkin-ring sign may be more than twice that without napkin ring sign (1.10 vs. 0.46 mm^2^) (53) corresponding to some studies' indications that a necrotic core area >1 mm^2^ when plaque is prone to rupture (14). The density of the ring is greater than that of the inner core but <130 HU in CT scans. Currently, the napkin-ring sign is considered to be a special CT feature of plaque with a large necrotic core, and it is a reliable marker of plaque instability (7, 14).

### High-Risk Plaque Features on CCTA in Prediction of Acute Coronary Syndrome

Although the risk of plaque instability increases with the degree of coronary stenosis, most of the culprit lesions found in ACS are considered non-obstructive before rupture. Previous studies indicated that the characteristics of high-risk plaques identified by CCTA have a prognostic value independent of stenosis and atherosclerotic burden (41) (see Table 1).

**Table 1:** Prediction of acute coronary syndrome by high-risk plaque CCTA features.

**Study author**	**Study method**	**Number of included patients**	**Type of CT equipment**	**Analyzed high-risk plaque features**
Hoffmann et al. (11)	ACS vs. SA	14/9	16-slice	Positive remodeling
Motoyama et al. (22)	ACS vs. SA	38/33	16-slice	Positive remodeling, low-attenuation NCP (<30 HU), and spotty calcification
Motoyama et al. (49)	ACS vs. non-ACS	15/1,044	16/64-slice	Positive remodeling and low attenuation
Kitagawa et al. (54)	ACS vs. non-ACS	21/80	64-slice	NCPs (<40 HU), positive remodeling and spotty calcification
Pflederer et al. (55)	ACS vs. SA	55/55	DSCT	Positive remodeling, lower attenuation and spotty calcification
Kim et al. (43)	ACS vs. SA	35/36	64-slice	Spotty calcification, low attenuation (≤60 HU) and positive remodeling (RI ≥ 1.05)
Ferencik et al. (42)	ACS vs. non-ACS	21/13	64-slice	Positive remodeling (RI > 1.05), low attenuation (<90/30HU) and spotty calcification
Otsuka et al. (34)	ACS vs. non-ACS	24/871	64-slice	Positive remodeling (RI > 1.1), low attenuation (<30 HU), and napkin-ring sign
Puchner et al. (10)	ACS vs. non-ACS	37/435	64/128/256-slice/DSCT	Positive remodeling, low attenuation (<30 HU), napkin-ring sign and spotty calcification
Motoyama et al. (8)	ACS vs. non-ACS	88/3,070	16/64/320-slice	Positive remodeling (RI ≥ 1.1), low attenuation (≤30 HU) and plaque progression
Chang et al. (41)	ACS vs. non-ACS	234/234	64-slice/DSCT/Other	Positive remodeling (RI ≥ 1.1), low attenuation (<30 HU) and spotty calcification
Williams et al. (45)	MI vs. non-MI	41/1,728	64/320-slice	low attenuation (<30 HU)

*ACS, acute coronary syndrome; CCTA, coronary computed tomography angiography; SA, stable angina; NCP, non-calcified plaque; HU, Hounsfield units; DSCT, dual-source computed tomography; RI, remodeling index; MI, myocardial infarction*.

In 2007, Motoyama et al. found that compared with SA lesions, the frequency of positive remodeling, non-calcified plaques or low-attenuation plaques (<30 HU) and spotty calcification on CCTA was higher in ACS culprit lesions, and these three characteristics could independently predict the occurrence of ACS. Moreover, among the three plaque features, positive remodeling has the highest accuracy in ACS prediction (22). In addition, an increase in the RI of culprit lesions in ACS patients compared with stable plaques in ACS and stable angina (SA) patients was also observed in other cross-sectional studies (11, 43, 54, 55).

In a subsequent study, Ozaki et al. compared the characteristics of lesions with intact fibrous caps and ruptured fibrous cap in ACS patients. The results revealed 88% of ruptured plaques had low-attenuation characteristics (*P* = 0.01) (56). These results were consistent with previous studies (43, 54, 55). Recently, a post-mortem multiple comparison analysis based on the SCOT-HEART study which included 1,769 patients with median follow up of 4.7 years identified the predictive effect of low-attenuation plaques on future cardiovascular events. They found that 4% was the cut-off value of low-attenuation plaque load as well as the strongest predictor of myocardial infarction. The predictive effect was independent of cardiovascular risk factors, calcification score and percentage of stenosis (45).

In a number of cross-sectional studies, investigators found that spotty calcification was associated with culprit lesions in ACS (49–52, 55). Compared with ACS, large calcification (≥3 mm) was more common in SA (22, 43). However, the predictive value of spotty calcification for plaque rupture is still controversial due to the huge heterogeneity in results (57). In addition, the definition and pathophysiological role of spotty calcification were questioned in studies using other imaging technologies such as OCT (58).

On CCTA, another manifestation of high-risk plaques is napkin ring sign. In a prospective study, Otsuka et al. recruited 895 patients and found that the hazard ratio (HR) of ACS in patients with the napkin ring sign was 5.55 [95% confidence interval (CI), 2.0–14.70; *P* < 0.001], which indicated napkin ring sign was an independent predictor of ACS (34). In a long-term follow-up study using ACS as one of the primary points, a strong prognostic implication of napkin-ring sign was also demonstrated by Feuchtner et al. (59).

Combining the characteristics of high-risk plaques could better predict the risk of ACS in the future (10, 42). The result of another study, which included 1,059 patients with follow-up for 27 ± 10 months also showed that PR or low-attenuation plaques, especially in combination, was independent predictors of future ACS events (49). Subsequently, this result was confirmed in a follow-up study (8). Furthermore, in the *post-hoc* analysis of the SCOT-HEART study, the adverse plaque was defined as a plaque with one or more of the features above observed. The results revealed that patients with adverse plaques had three times higher risk of myocardial infarction than patients without adverse plaques, and the prediction effect was stronger when combined with the presence of stenosis (23).

Although there remain some controversies regarding the definition of high-risk plaques on CCTA and the predictive efficacy of different high-risk plaque characteristics in future ACS events to date (5, 8, 25, 41, 60–63), the correlation between the existence of high-risk plaques and the occurrence of future ACS events is generally obtained.

## Emerging CCTA Technologies and Acute Coronary Syndrome

The emerging technologies have promoted the clinical application of CCTA. Right now, CCTA can not only identify the morphological characteristics of high-risk plaques non-invasively, but also enable the intracoronary and extracoronary imaging, so as to realize the comprehensive assessment of high-risk plaques and surrounding environment, which may provide personalized risk assessment of future ACS events and further guide clinical decision-making (12).

### Computational Fluid Dynamics and Acute Coronary Syndrome

Plaque rupture happens when the stress inside the plaque exceeds its strength (64), while rupture of plaques is a complex biomechanical process, which is affected by the plaque structure and composition, external forces, and hemodynamic factors. Even if the plaque manifests the same vulnerable characteristics, the risk of rupture is different due to the heterogeneity of hemodynamic press exerting on the plaque (13, 65). Therefore, in addition to the assessment of plaque characteristics, accurate evaluation of plaque hemodynamic parameters is also vital for the identification of high-risk plaques and the prediction of ACS risk.

**Figure 3:**
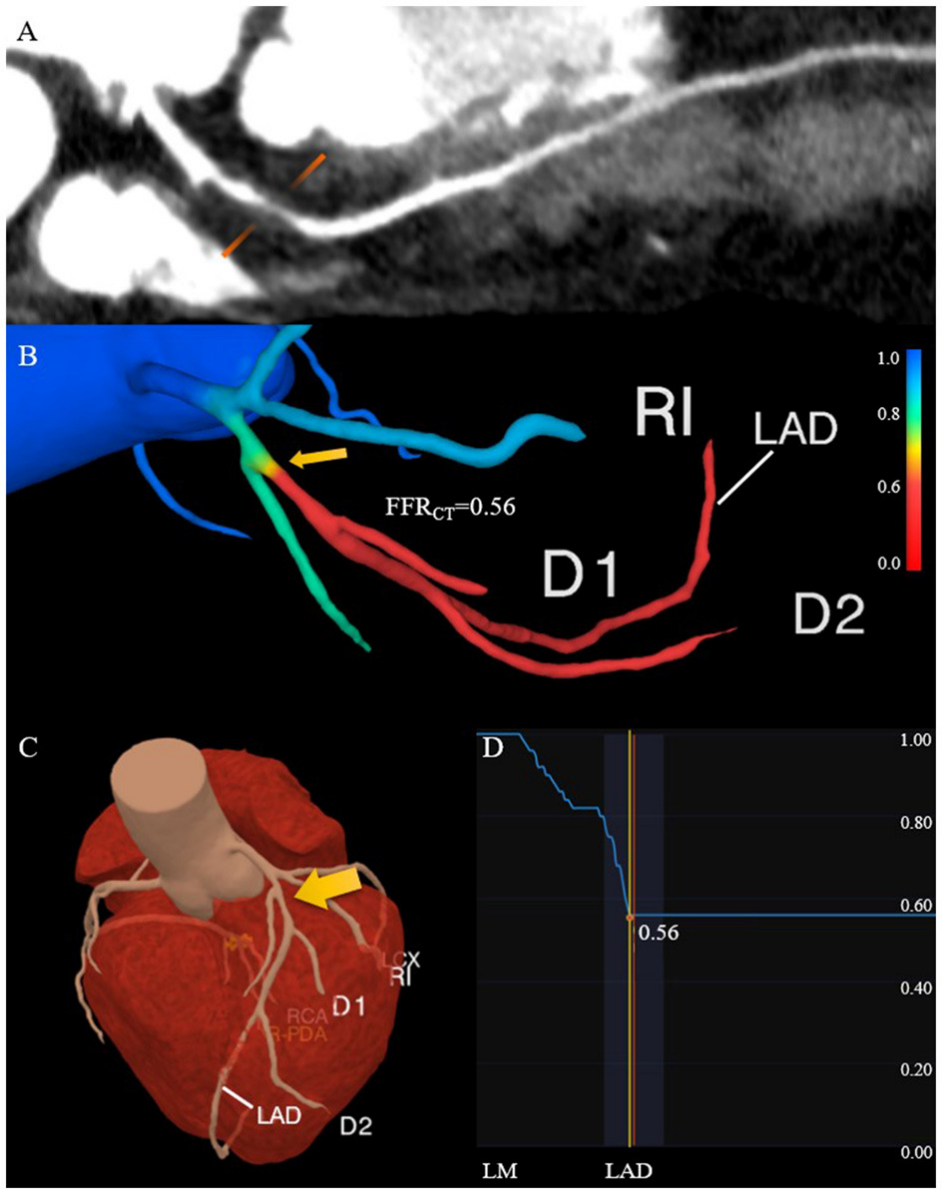
Comprehensive assessment of the characteristics of high-risk plaque with CCTA. **(A)** The lesion in the proximal of the left anterior descending coronary artery has positive remodeling and low attenuation. **(B)** The FFR_CT_ map derived from computational fluid dynamics shows a value of 0.56 distal to the stenosis, which indicates lesion ischemia. The transition from blue to red along the left anterior descending coronary artery indicates the decreasing trend of the FFR_CT_. **(C)** The 3D model diagram of the coronary artery, and the yellow arrow indicates the location of the high-risk plaque. **(D)** The curve of the FFR_CT_ value from the proximal to distal of the left anterior descending branch. CCTA, coronary computed tomography angiography; FFR_CT_, fractional flow reserve derived from CCTA.

Fractional flow reserve (FFR) is the ratio of the pressure at the distal end of the stenosis to the pressure at the proximal end of the normal vessel in a state of maximum hyperemic condition. In recent years, FFR derived from CCTA (FFR_CT_) has used advanced fluid dynamics analysis method which combines the advantages of CCTA and traditional FFR (Figure 3). It is an image post-processing technology that applies hemodynamics to CCTA examination, which uses conventional standardized CCTA image data to evaluate the hemodynamic differences of coronary artery stenosis (66). Assuming that blood is an incompressible Newtonian fluid with constant density and viscosity, the flow and pressure in the coronary model volume can be calculated by the Navier-Stokes equations (12, 58). Three prospective clinical trials (67–69) all have verified that FFR_CT_ can evaluate coronary artery stenosis from both anatomy and function. In addition, it has avoided the invasive operations of traditional FFR and the complications such as coronary artery tearing, bleeding, arrhythmia, myocardial infarction, and so on (70). This “one-stop” technology can fundamentally avoid unnecessary coronary angiography and revascularization. Moreover, it can provide more information for clinical practice and has the potential to replace other traditional methods recommended in clinical guidelines. As a long-term gatekeeper to guide revascularization, FFR_CT_ is a new hot spot in clinical research (69, 71, 72).

FFR_CT_, change in FFR_CT_ across the lesion (ΔFFR_CT_), wall shear stress (WSS) and axial plaque stress are important hemodynamic parameters, which has been reported to be associated with the occurrence of adverse clinical events (13, 65, 66, 73–75). Lee et al. had demonstrated the role of these non-invasive hemodynamic parameters in identifying high-risk plaques leading to ACS by comparing adverse plaque characteristics and non-invasive hemodynamics parameters between culprit and non-culprit lesions (76). The adverse hemodynamics were defined as FFR_CT_ ≤ 0.80, ΔFFR_CT_ ≥ 0.06, WSS ≥ 154.7 dyn/cm^2^ or axial play stress ≥ 1606.6 dyn/cm^2^. The results showed that the FFR_CT_ of culprit lesions was lower compared with non-culprit lesions, which was consistent with the findings of Ferencik et al. (77). While ΔFFR_CT_, WSS, and axial plaque stress are higher in culprit lesions, and ΔFFR_CT_ has the highest incremental value. Moreover, Park et al. proved that ΔFFR_CT_ has a powerful value in ACS risk prediction (78), indicating that lesion-specific hemodynamic parameters have greater influence on the occurrence of plaque rupture and ACS than that in vascular level. The value of WSS in identifying high-risk plaques has also been confirmed (79–81).

Compared with plaques with high-risk anatomical characteristics or high-risk hemodynamic characteristics, plaques with both characteristics have a significantly higher risk of progressing to ACS (HR: 3.22; 95%CI: 1.86 to 5.55; P < 0.001; and HR: 11.75; 95%CI: 2.85–48.51; *P* = 0.001, respectively) (76). These studies are very valuable because they identified adverse hemodynamic characteristics before the onset of ACS. On the basis of anatomic severity and adverse plaque characteristics, the non-invasive evaluation of hemodynamic parameters was added, which improved the C-index and reclassification ability in identifying the high-risk plaques that led to ACS. In the future, the evaluation of non-invasive hemodynamic parameters may improve the identification of plaques prone to rupture as well as the prediction efficiency of high-risk plaques for ACS, and help to make the risk stratification. Further studies need to explore and determine the optimal hemodynamic parameters or combination of different patients and lesion subgroups.

### Pericoronary Fat and Acute Coronary Syndrome

**Figure 4:**
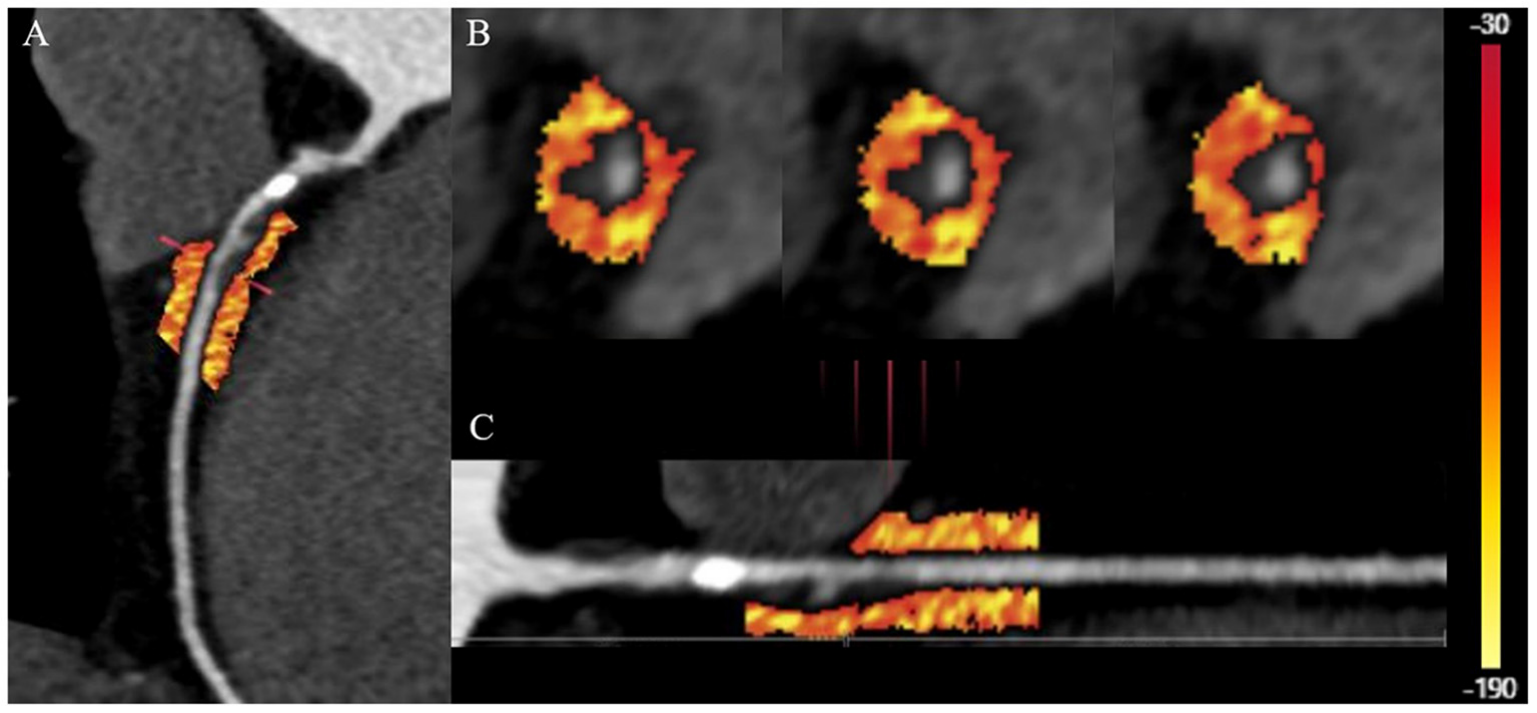
Quantification of PCAT CT attenuation of low-attenuation plaques in the proximal-mid right coronary artery. **(A)** The multiplanar reconstructed image of PCAT measured with the range from −190 to −30 HU. **(B)** Cross-section images of PCAT measure. **(C)** Straightened image of PCAT measure. PCAT, pericoronary adipose tissue; HU, Hounsfield units.

Vascular inflammation can promote the progression of coronary atherosclerosis and vulnerable plaque rupture, leading to the occurrence of ACS (64). Epicardial pericoronary adipose tissue (PCAT) is a special type of adipose tissue. It interacts with the adjacent vascular wall, regulates the cardiovascular biological function in a paracrine manner, and changes its phenotype in response to signals from vascular walls (82–86). Antonopoulos et al. (84) studied the gene expression, histology and CT imaging of adipose tissue samples collected during cardiac surgery, and considered that the CT density of adipose tissue (usually defined as −190 to −30 HU) reflects the balance of lipid and water phase, which is a marker of adipocyte size and lipid content. Inflammatory signals released by inflamed blood vessels are directly spread to PCAT, which can induce local lipolysis and inhibit fat formation, and also increase microvascular permeability, thus promoting perivascular edema. With the decrease of lipid content and morphology in adipocytes of PCAT, the lipid phase in adipose tissue decreases and the water phase increases, resulting in different gradients of adipocytes around the coronary artery. A further study confirmed that PCAT CT attenuation measured by CCTA can detect vascular inflammation confirmed by biopsy, and the fat attenuation index (FAI) was proposed (84). Pericoronary FAI was used to track and quantify the composition changes of PCAT by evaluating the spatial changes of peripheral fat attenuation by CCTA, which was the average density of adipose tissue in the target area (84) (Figure 4), reflecting inflammatory burden of target coronary segment (A higher pericoronary FAI was associated with a higher inflammatory burden).

Inflammation of adipose tissue around the plaque will directly affect the formation and stability of coronary plaque. Therefore, PCAT attenuation measured by CCTA is a promising indicator for identifying high-risk plaques. In patients with ACS, cases of PCAT stranding have been reported around culprit lesions (87). Goeller et al. retrospectively recruited 19 patients with ACS and 16 patients with stable CAD (88). They found that culprit lesions were associated with increased FAI around the lesions. The frequency of pericoronary FAI ≥-68.2 HU of culprit lesions in ACS is higher, which can be used as a potential cut-off value to distinguish culprit lesions and non-culprit lesions. Therefore, combining the characteristics of high-risk plaques with the FAI around the plaques can be more reliable in identifying the high-risk plaques leading to ACS. Goeller et al. further studied the correlation between pericoronary FAI and coronary plaque progression (89). They found that the increased non-calcified plaque burden was related to the increased FAI, on the contrary, the decreased non-calcified plaque burden was related to the decreased FAI, and FAI ≥ −75 HU around the proximal end of right coronary artery (RCA) was an independent predictor of increased load of non-calcified plaques and total plaques. Therefore, PCAT is helpful to identify patients with high risk of plaque progression.

Although FAI can detect the changes of PCAT composition caused by coronary artery inflammation, it can only measure the average density of the ROI and cannot fully reflect the fine structural characteristics of the ROI. Inflammation, fibrosis, and angiogenesis are three main causes of adipose tissue dysfunction (90). Advanced imaging omics analysis can supplement the deficiency of FAI, revealing the structural changes of PCAT that cannot be recognized by the naked eye, so as to carry out more personalized assessment, and finally provide new biological insights into the pathogenesis of the disease. Recently, some researchers have verified the feasibility of PCAT image transcriptomics (91). They collected adipose tissue biopsies from 167 patients undergoing CCTA and cardiac surgery. In addition, they used image transcriptomics to correlate the gene expression of inflammation, fibrosis, and microvascular remodeling with the image omics features extracted from CT images. They proposed a new machine learning derived biomarker-fat radiomic profile (FRP), which was trained and validated to discover the PCAT imaging features related to inflammation, fibrosis, and microvascular remodeling associated with an increased cardiovascular risk. The FRP turned out to be affected by a series of factors such as scanning conditions, and the analysis was time-consuming (91). At present, a new application is being developed to automatically detect the coronary artery and pericardium, and may realize pericoronary space segmentation and feature extraction, so as to calculate FRP and FAI, and simultaneously correct the technical and anatomical information. The calculation time can be reduced to 5 min. In conclusion, PCAT imaging analysis is a new field, and its application in assessing the risk of ACS needs to be explored.

## Limitations and Future Directions of CCTA in Acute Coronary Syndrome

Modern CCTA assessment provides a series of advantages by combining the coronary anatomy, plaque morphology, atherosclerotic plaque load and coronary blood flow, which allows for the evaluation of morphological and functional characteristics of high-risk plaques, and the individual risk of ACS.

In addition to CCTA, positron emission tomography (PET), magnetic resonance coronary angiography (MRCA), IVUS, OCT, and other imaging modalities are also effective methods to assess coronary atherosclerotic plaques (92–98). Notably, CCTA has irreplaceable advantages over other examinations. Firstly, CCTA can quickly provide powerful diagnostic information. Secondly, compared to coronary angiography, IVUS, and OCT, CCTA is a relatively non-invasive method with low requirements for practitioners and high universality of clinical application. In addition, CCTA has a higher spatial resolution in identifying high-risk plaque characteristics and more stable image quality than MRCA and PET. With the development of advanced hardware technology, such as photon count detectors and novel contrast agents, potential applications of multi-parameter techniques may make the plaque characterization more precise. Moreover, in previous studies, CCTA has been confirmed as a cost-effective imaging strategy (99–103).

However, there still remain certain drawbacks in CCTA. Compared with IVUS and OCT (92–98), CCTA has relatively lower spatial resolution, which hindered the detection of microscopic structure in histology (9, 104), such as the fibrous cap thickness or plaque rupture. Further CCTA studies are needed to investigate pathophysiology of rapid progression of high-risk coronary plaques leading to ACS, which will offer clinical utility on the management of patients with CAD. Recent development of intravascular imaging enables to evaluate biomechanical plaque rupture stress *in vivo*, offering prognostic implication of patients at higher risk of ACS (105, 106). The combination of intravascular imaging and CCTA may help development a novel methodology for plaque assessment of the probability of coronary plaque rupture. In addition, the beam hardening and related halo caused by the high attenuation structure may affect the image quality (107–109), causing reduced accuracy when identifying heavily calcified lesions. Moreover, the temporal resolution of CCTA can be affected by heart cycle as well as respiration, and it is not uncommon to find the motion artifacts in images of CCTA. Another limitation is the potential increased radiation exposure (110). While recent development of CCTA enables to reduce radiation doses by increasing the use of low-end potential scans, high-pitch scan protocols, and iterative image reconstruction. The true cardiac-capable photon-count detector will be likely available soon (111–113). The emergence of wide-detector technology, dual source X-ray and high-pitch acquisition platforms has made it possible for end-diastolic and end-systolic acquisition in a single heartbeat and with sub-millisievert dose (114–116).

Although the presence of high-risk plaque features has been widely recognized in the prediction of clinical ACS, its positive predictive value for ACS is still limited (60). In previous studies, there was a relatively long-time span between CCTA and ACS events, so the changes in drug treatment during this period have considerable heterogeneity. In addition, the clinical significance of these findings may be uncertain in the case of patients having received the effective medication. Therefore, these high-risk plaque characteristics should be further investigated in cohort studies and prospective ACS prevention trials. In addition, multicenter randomized controlled trials are needed to determine whether drug treatment and/or intervention based on high-risk plaque CCTA characteristics can improve the clinical outcomes of patients.

Recently, some technological innovations in image acquisition, post-processing and other imaging biomarkers have become more and more important, which may affect the implementation, interpretation and clinical application of CCTA. To achieve high image quality should continue to be emphasized in the future, so as to accurately apply new methods such as functional assessment and plaque quantification to CCTA imaging (117). Moreover, artificial intelligence and machine learning methods may get more attention (118–120). Currently, emerging technologies such as CCTA-based identification of high-risk plaque features and FAI have not been verified in randomized controlled trials. Therefore, in the future, further studies are needed to prove the clinical benefits of CCTA application. Furthermore, with the development of hardware and advanced analysis tools, and true clinical and cost-effectiveness data, it will continue to popularize in clinical practice (121).

## Conclusion

Current findings support more attention and careful management of patients with high-risk atherosclerotic lesions, and monitoring plaque progression for individualized treatment. CCTA has an important significance in research and daily clinical practice, which allows for not only predicting the occurrence of ACS by analyzing the characteristics of high-risk plaques but also improving the predictive value in future ACS through emerging technologies such as computational fluid dynamics and evaluation of pericoronary radiation group characteristics (such as pericoronary FAI). Furthermore, combining multi-dimensional methods can comprehensively evaluate the anatomy and biology of the coronary artery, and further integrate CCTA into clinical practice. However, the identification of high-risk plaque characteristics based on CCTA and emerging technologies such as FAI have not been verified in randomized controlled trials with definite results, which requires verification of the clinical benefits of CCTA applications in the future.

## Author Contributions

GL substantially planned, wrote, and revised the manuscript and made illustrates and the table. WY polished and revised the manuscript. JO and XL contributed to the collection of CT images. ZT and TL contributed to linguistic revision of the manuscript. HL substantially contributed to instruction of the writing and revision of the manuscript. All authors read and approved the final manuscript.

## Funding

The work was supported by the Key R&D Program of Guangdong Province of China (No. 2018B030339001 to HL), the National Natural Science Foundation of China (No. 81974262 to HL), Guangdong Basic and Applied Basic Research Foundation (No. 2020A1515010650 to HL), and Guangdong Nurses Association Research Foundation (No. gdshsxh2021b077 to XL).

## Conflict of Interest

The authors declare that the research was conducted in the absence of any commercial or financial relationships that could be construed as a potential conflict of interest.

## Publisher's Note

All claims expressed in this article are solely those of the authors and do not necessarily represent those of their affiliated organizations, or those of the publisher, the editors and the reviewers. Any product that may be evaluated in this article, or claim that may be made by its manufacturer, is not guaranteed or endorsed by the publisher.
